# Practical guide to the use of digital slides in histopathology education

**DOI:** 10.1136/jcp-2024-209415

**Published:** 2024-03-28

**Authors:** Bethany Jill Williams

**Affiliations:** 1 National Pathology Imaging Co-Operative, Leeds Teaching Hospitals NHS Trust, Leeds, UK; 2 University of Leeds, Leeds, UK

**Keywords:** Education, Medical, Histology, Medical Informatics Computing

## Abstract

Digital pathology (the technology whereby glass histology slides are scanned at high resolution, digitised, stored and shared with pathologists, who can view them using microscopy software on a screen) is transforming the delivery of clinical diagnostic pathology services around the world. In addition to adding value to clinical histopathology practice, digital histology slides provide a versatile medium to achieve the educational needs of a variety of learners including undergraduate students, postgraduate doctors in training and those pursuing continuing professional development portfolios. In this guide, we will review the principal use cases for digital slides in training and education and I will share tips for successful use of digital pathology to support a range of learners based on experience gathered at Leeds Teaching Hospitals National Health Service Trust and the National Pathology Imaging Co-Operative during the last 5 years of digital slide usage.

## Background

Traditionally, histopathology education has been delivered using glass slides, conventional light microscopy and physical textbooks with static photographs of classic disease presentations. Teaching sets of glass slides are accrued by universities and clinical departments, and shared with students, who view the slides on individual microscopes or via a multiheaded microscope with a trainer.^1 2^ Many of these slides are unique training resources, containing precious patient tissue that cannot be recut or duplicated, which places major constraints of the availability and transferability of the slides. Use of a conventional light microscope requires practice, experience and development of core visual and motor skills, and can form a stumbling block for efficient histopathology education of a novice microscopist.^3^

## What is a digital slide?

**Figure 1:**
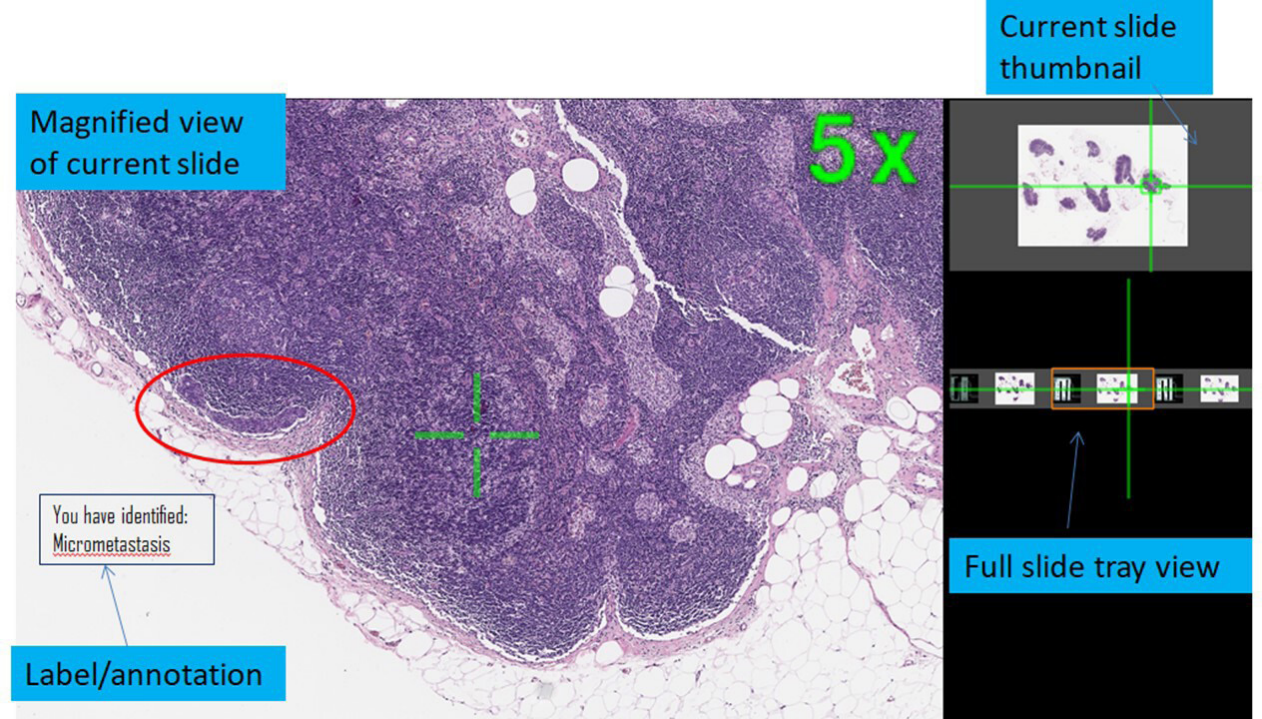
An example of a digital slide from the Leeds Teaching Hospitals National Health Service Trust digital training archive.

A digital slide contains digital information equivalent to hundreds of light microscope high-power fields of view. Multiple digital images are stitched together to create a seamless ‘whole slide image’ which is stored in a pyramidal file format that can be scanned in the x and y axis, and viewed dynamically through a range of magnifications (typically from 0× to 60× equivalent magnification). Digital slide viewing software can be integrated with image editing software that facilitates digital measurement, overlay drawings, superimposed annotations and accompanying text, so that learners can view trainer-authored didactic labels and text, or add their own notes to an image (see figure 1 for an example of an annotated digital slide.)^4^ Each digital slide can be viewed by multiple learners simultaneously and accessed from anywhere in the world.^2^

**Figure 2:**
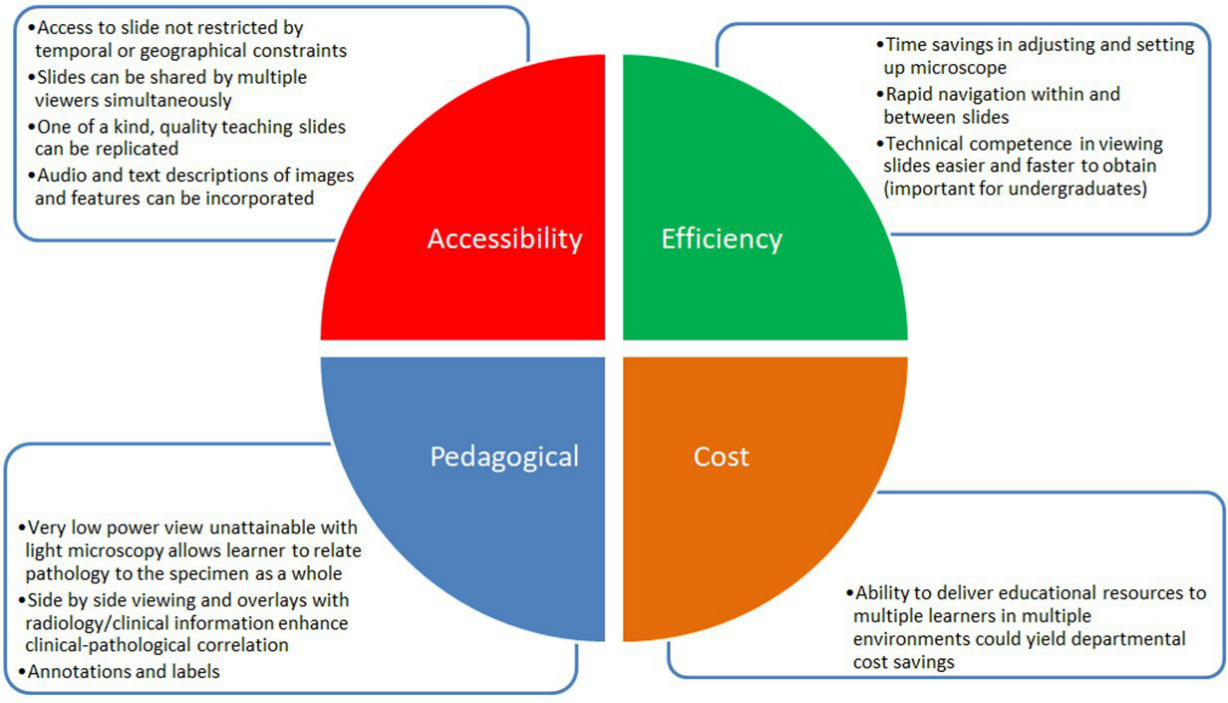
Key advantages of digital slides versus glass slides in histopathology education.

The flexibility of the digital slide medium versus the glass slide has a number of key advantages in terms of accessibility, efficiency, pedagogy and cost, which are summarised in figure 2. The principal disadvantage, which is particularly relevant for postgraduate learners (histopathology doctors in training), would be difficulties in obtaining key competencies in light microscope use if digital slides are the exclusive method of curriculum delivery.^2^

## Digital microscopy in undergraduate curricula

The place of microscopy, and indeed, of histopathology, in undergraduate medical curricula has evolved over the last few decades. Up until the early 1990s, most taught pathology modules and courses were reliant on a combination of didactic lectures and pathology case-based exercises centred on conventional light microscopy.^3 5^ A number of curricular reforms have shifted this paradigm:

1. A decrease in time allotted to pathology and basic sciences teaching in favour of increased exposure to clinical environments throughout undergraduate programmes.
2. A shift from departmentally organised didactic teaching towards integrated and systems-based curricula, with more emphasis on case-based and problem-based learning and self-directed study.
3. Augmentation of conventional light microscopy with digital microscopy techniques in standard clinical practice.
4. Loss of multimicroscope ‘dry’ laboratories in medical schools in favour of multipurpose computer laboratories that can be used for a wider range of educational activities.

Digital slide technology has been progressively integrated into undergraduate medical, dental and allied health science curricula since the early 1990s, with digital microscopy laboratories, accessed via personal devices or in medical school-based computer laboratories gradually replacing fixed training facilities equipped with boxes of glass slides and standard optical microscopes.^6–10^ Digital slides with links to additional resources, including gross macroscopic photography, radiological images and further study material, enrich the learning experience. In addition to replacing the microscopy room of pathology-rich courses, micro-doses of digital slide exposure, in the form of use of digital slides within lectures and PowerPoint (Microsoft) slide sets, allow inclusion of histopathology content in courses that may not have previously included microscopic material. Students viewing digital slides have been demonstrated to spend more time interacting with histopathology material, and perform as well or better in evaluations than their peers in fixed microscopy laboratories.^6–10^

Aside from large-group ‘virtual laboratory’ teachings, digital slides have been successfully incorporated into small-group learning settings too. University of Washington students used PathPresenter software (https://pathpresenter.net/) to preview curated digital slide sets, which were then discussed in group settings.^11^ Digital slides were used as part of case-based activities in which students were given a patient presentation and then worked the slides up virtually, ordering laboratory tests, imaging and biopsies, and receiving results in real time. These small-group activities encouraged increased participation and helped foster a sense of community.

For medical students interested in histopathology careers, an elective period of study in a hospital pathology department is often incorporated into the final years of an undergraduate degree. Cancellation of in-person attachments during the COVID-19 pandemic in the USA resulted in the creation of online ‘virtual clerkship’ placements for medical students in Seattle.^12^ Use of a digital slide viewing platform opened up these learning opportunities to an unprecedented number of candidates, located in institutions beyond state boundaries, and demonstrated an unanticipated level of demand and interest in histopathology, a subspecialty which is frequently under-recruited at postgraduate level. Students were able to attend consultant pathologist ‘sign-out’ sessions via Zoom (Zoom Video Communications), during which faculty shared digital slide images. The Zoom annotation functionality permitted users to draw on the screen, so educators can circle or point out key features, and can ask students to demonstrate findings, or indicate parts of the slide that are puzzling them.

## Digital slides in postgraduate education

Digital slide technology has experienced a more mixed reception in the realm of postgraduate medical education. This is largely due to the lag in adoption of digital pathology for primary diagnostic practice in clinical settings. Practice differs widely from country to country and from city to city in terms of access to and usage of digital slide images for histopathology service delivery, and many departments still depend on the use of glass slides and conventional light microscopy. Proficiency in digital pathology techniques is still not mentioned in the Accreditation Council for Graduate Medical Education Milestones requirements for trainees,^13^ and is not a core component of Royal College of Pathologists postgraduate curriculum for histopathology.^14^ The rationale for this omission is that trainees still need to balance use of digital slide technology with hands-on experience of traditional microscopy and acquisition of technical skills for glass slide diagnosis including Koehler illumination. A number of online digital slide resources are available to support the learning needs of postgraduate trainees, which are summarised in table 1.

**Table 1:** Digital slide resources for histopathology trainees

Resource	Description	Link
Leeds Virtual Microscopy	Searchable database of digital slides, previous postgraduate examination cases	https://www.virtualpathology.leeds.ac.uk/
MGH Pathology	General pathology and frozen section digital slides	https://learn.mghpathology.org
Juan Rosai Collection	General pathology digital slides	https://www.rosaicollection.org/
University of Michigan	General pathology digital slides	https://www.pathology.med.umich.edu/apps/slides/
University of Oklahoma	Digital slide atlas, digital slide quizzes, case of the month	https://www.ouhsc.edu/pathologyJTY/OUMC/Default.htm
DAPA	Digital slide case of the week; requires DPA membership free to trainees	https://digitalpathologyassociation.org/digital-anatomicpathology-academy
Pathology Portal (Royal College of Pathologists, UK)	Online, interactive educational resources including digital slide-based resources; requires Learning Hub registration, free to access	https://rcpath.org/profession/pathology-portal.html

All URLs accessed on 17 December 2023.

DAPA, Digital Anatomic Pathology Academy; DPA, Digital Pathology Association; MGH, Massachusetts General Hospital.

In departments that do not use digital slides as part of clinical practice, shared educational slide collections curated on the web can prove a valuable resource, while in departments with an evolving clinical archive of ‘live’ patient cases, trainers are able to direct trainees to the most relevant digital cases for that specific trainee. They are also able to share their current ‘real-world’ workload with the trainee, without delaying final diagnosis for the patient, as both trainee and trainer can review the same case synchronously, on separate workstations, and share their impressions of the case during dedicated feedback and discussion sessions.^1^

## Digital slides in continuing professional development

The underlying aim of continuing professional development (CPD) is to enable pathologists to keep up to date with current knowledge and advances in their field, obtain professional revalidation and continue to practise safely. Traditionally, pathologists attended conferences, read journals, viewed online lectures or took courses to obtain the recommended CPD credits each year. Digital slides have provided a practical alternative and allow practitioners to earn CPD credits from their homes. Topography specific, online CPD events stream digital slides online, and have successfully incorporated digital slide-based pre-event and post-event self-assessment cases to consolidate learning.^1^ In the UK, external quality assurance materials have been shared in the format of digital slides, hosted by the University of Leeds Virtual Pathology website (https://www.virtualpathology.leeds.ac.uk/eqa), allowing hundreds of pathologists to share rare, informative cases for discussion and consensus review.

## Top tips for incorporating digital slides into histopathology training and education

### Create digital slide cases that are authentic and appropriately challenging

**Figure 3:**
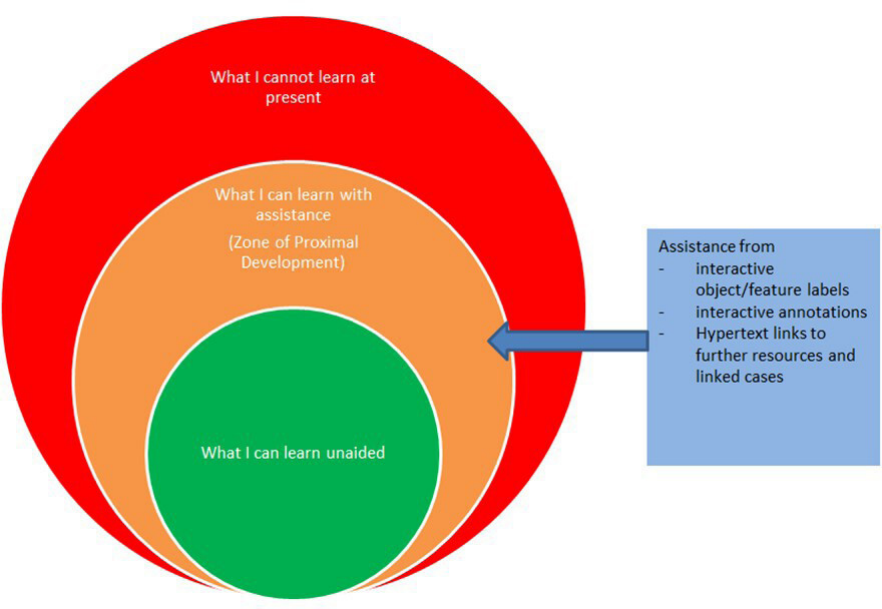
Scaffolding in the zone of proximal development using digital slide-enabled features. Adapted from Vygotsky.^15^

To be effective, educational digital slides should be interesting, challenging and authentic, and care should be taken to ensure that the level of difficulty and complexity of cases match the learning needs of participants. For the purposes of medical undergraduate education, clear, unambiguous examples of common pathologies should be presented, with suitable interactive labels and annotations to aid identification of key features. More advanced learners, including pathologists in training, should be presented with cases of increasing rarity and difficulty. They also need to review an appropriate number of ‘normal’ slides, and slides with dual pathologies or unusual features. Table 2 documents how digital slide use is tailored to different learner groups (undergraduates, trainees and CPD users) at Leeds Teaching Hospitals National Health Service (NHS) Trust. Tuning the level of difficulty of the digital slides allows the learner to explore their personal zone of proximal development^15^—the difference between what they can achieve without the ‘scaffolding’ of prompts of annotations and what they can do with this extra assistance (see figure 3). Wass and Golding have demonstrated that this process of scaffolding leads to increased learning,^16^ and it also allows for a more personalised, bespoke approach to learning, where the learner can choose the amount of scaffolding they require in different scenarios, and opt to hide or reveal different levels of labelling and annotation. Similarly, trainers can enable or block signposts, labels and annotations for different users of the same digital slide, so that multiple learners, with differing levels of ability and confidence, and occupying different zones of proximal development, can share the same educational cases.

**Table 2:** Use of digital slide cases by different learner groups at Leeds Teaching Hospitals NHS Trust

	Types of case	Enhanced digital features	Types of learning experience
Undergraduate	Unambiguous examples of common pathologies Curated, anonymised digital slide sets	Interactive labels and annotations Hypertext links to further resources including glossary of terms	Didactic lectures Small group/special study module teachings Self-study material (digital textbook)
Postgraduate (pathologist trainee)	More complex pathologies, typical and non-typical examples, inclusion of laboratory and digital artefact A mixture of curated anonymised ‘training’ slide sets and live cases	Interactive labels and annotations Hypertext links to other digital slides illustrating the same pathology	Didactic lectures/large-group teaching sessions Small-group and individual teaching
Continuing professional development	Challenging areas of diagnosis with high levels of intraobserver variability in diagnosis	Screen sharing with remotely situated experts	External quality assurance schemes Intradepartmental and interdepartmental case discussion

NHS, National Health Service.

### Relate digital slides back to the clinical context

Small-group teaching in undergraduate histopathology education revolves around case-based learning (CBL), where the digital patient is represented by written case study including demographic information and clinical history, possibly accompanied by radiological findings, similar to the approach in other medical specialties.^17^ Digital slides are easily incorporated into these cases and allow students to gain ‘hands-on’ experience navigating through tissue to identify pathologies. The only prerequisite is access to a laptop or desktop computer and a high-speed internet connection, so CBL can be conducted remotely (with the group connected via Microsoft Teams or similar software, using shared desktop functionality), or in person using information technology (IT) laboratory facilities for larger groups, or a shared computer workstation with a large screen for smaller groups. CBL has been shown to promote the development of analytical thinking skills by engaging students in discussion about complex, real-life scenarios.^18^ Typical group size might be 6–10 participants, although this type of exercise can be adapted for smaller or larger groups. A facilitator should be on hand to guide the group as the students apply knowledge garnered from self-study and didactic elements of the undergraduate curriculum to new situations. The role of the facilitator here is to correct, redirect and provide feedback. Students trained using problem-based learning techniques have been demonstrated to develop higher levels of critical thinking compared with students adhering to a more traditional lecture-based curriculum.^19 20^

**Figure 4:**
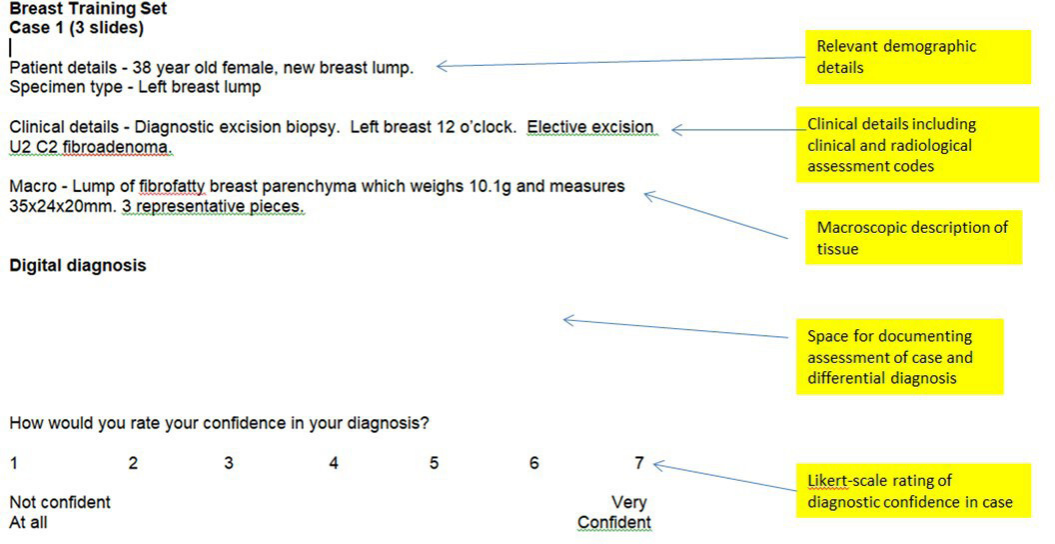
Example of a worksheet to accompany a digital slide case for a histopathology trainee used at Leeds Teaching Hospitals National Health Service Trust (written by the author of this manuscript).

CBL can also be used for postgraduate training of histopathology trainees, where it can be useful in small-group teaching settings to allow students to explore clinic–pathological correlation and how the medical context can affect the histopathological differential diagnosis. Digital slides should be presented alongside relevant medical history and multidisciplinary assessment (see figure 4). Unlike undergraduate students, postgraduate students in histopathology departments have to conduct the majority of their training individually, using self-directed techniques. CBL-type cases can be adapted to suit this training environment, with case lists and digital slide links circulated to be reviewed by learners individually and fit their clinical timetable. Cases can then be discussed at regular group meetings, led by a facilitator who ensures the most salient features of the case have been appreciated by the group, and leads discussion of ambiguous aspects of the clinical context and differential diagnosis. Cases should come accompanied by information that mimics the quality and quantity of information (or lack of it!) provided in clinical requests for histopathology assessment, and should use real-world nomenclature, acronyms and coding systems commonly used in local clinical practice. As histopathology trainees are super-numerary, and do not sign out cases independently while in training, it can be useful to ask trainees to rate their confidence in their diagnosis and assessment of a case. This gives an indication of how certain the trainee is in their skills, and how close they are to achieving sufficient comfort with a specimen or case type to independently sign out a diagnosis to a patient. These confidence ratings can be reviewed with the trainee’s educational supervisor to gauge whether a trainee is underconfident or overconfident, and to track progress with regard to particular specimen types.

### Aim for shared understanding and peer-to-peer learning

Vygotsky’s theories state that the process of knowing is affected by other people and is mediated by culture and community.^15^ It follows that all learning is a collaborative process, dependent on interactions with other members of the community. When using digital slides, learners are encouraged to build upon their existing knowledge of light microscopy if applicable, and transfer this to digital techniques, noting differences, challenges and improvements with the two different diagnostic media. When leading a group digital slide-based learning session, it can be helpful to encourage all members of the group to share their current level of experience and comfort with digital slide use. Sharing of knowledge and perspectives in this way can help individual participants construct their understanding of both the cases being discussed and the digital slide medium that is being used.

Postgraduate and continuing medical education in histopathology is often facilitated via formal or informal Communities of Practice (CoP)—professional groups with similar experiences, beliefs and values.^21 22^ In histopathology, these groups are generally organised around anatomical topographies of specialisation (for example, neuropathology, gynaepathology). These groups are likely to contain established specialist consultants alongside trainees with special interests in a particular subspecialisation. The sharing of digital slides from educational collections, quality assurance sets and everyday practice could form a key part of CoP interaction, via informal referrals and second opinion requests. The use of digital slides rather than glass slides for these types of activity is far easier and quicker, especially to engage with colleagues from separate institutions, and lowers the threshold for such interactions occurring. Interestingly, use of the digital slide rather than the glass slide medium here might alter the hierarchies within the CoP. More senior diagnosticians may engage in more ‘peripheral participation’^21^ if they lack confidence or experience in the use of specialised software, and may benefit from exploring enhanced software functionality with some of their more junior colleagues, while junior colleagues in turn can observe the diagnostic process through the eyes (and mouse clicks!) of an expert diagnostician. In this manner, both groups can also operate in their zones of proximal development, enhancing IT and diagnostic skills, respectively.

### Encourage reflective practice

The integration of theory and practice is one of the key tenets of healthcare education, and reflective practice is one of the key mechanisms by which this can be achieved. Learners of all stages should be encouraged to consider how they have engaged with the digital slides and what they have learnt. This ‘reflection-on-action’^23^ allows learners to cement their understanding and develop their knowledge base. Such reflections may form the basis of annual appraisal portfolio entries for consultants and trainees. Reflective work often works best as a collective and collaborative activity, where diverse insights and perspectives can help shape collective values. This fits well with the concept of the CoP, as discussed previously. While working through digital training slides, learners can reflect on their current diagnostic skills—this is reflection-in-action. The learner is conscious of the diagnostic tasks they are performing, and additionally aware of how they are completing the task and the knowledge that underpins the skill. The learner can create their own annotations on digital slides to document and reinforce this awareness of the diagnostic pathway they are following, and in this way they can move from superficial to deep learning.^24^

### Be creative in how you use digital slides, and the educators and learners you involve

The flexibility and transferability of the digital slide open up new opportunities for broadening the scope of learners and educators you involve in your teaching sessions. Learners from different regions, with similar specialist learning needs, can be connected and share tailored sessions with experts from anywhere in the world! In this way, we can improve equity of access to training opportunities for junior pathologists, who are no longer limited to the training staff and materials located in a single pathology department.

Similarly, the use of digital slide training sessions which can be accessed remotely can support the needs of trainees and learners who need to work and train more flexibly. At Leeds Teaching Hospitals NHS Trust, we were able to maintain training throughout periods of COVID-19 lockdown, and offer learning opportunities to trainees and trainers who were having to isolate, shield or take on increased caring responsibilities during the pandemic.^25^ One approach would be for trainers to assign links to digital slide cases and meet with small groups of trainees via Microsoft Teams meetings to share desktops, demonstrate how they approached the case and discuss their diagnoses. Trainers are then able to take control of the trainee case and redirect attention to particular features where necessary. The rapid movement from face-to-face to digital training necessitated by the restrictions of the pandemic period brought challenges, but has also caused many clinician educators to open up to the potential and opportunities afforded by remote and technology-enabled learning.

### Don’t be overly reliant on digital slides!

Digital slides offer a practical and creative approach to histopathology training, but it is important to remember that they should be used as an adjunct to, rather than a replacement for, immersive training using standard glass slide workloads and workflow. Authentic, real-world workplace-based learning, centred around shadowing a consultant trainer and exposing the trainee to some of the more pragmatic issues around contributing to a live clinical service, is a vital part of postgraduate histopathology training. This is the best environment for what Eraut describes as the implicit and tacit learning that occurs in the absence of overt teaching. In histopathology practice,^26^ this includes knowledge of contexts and organisations acquired through socialisation and participation. These include understanding of people and situations. The ideal training programme incorporates a range of real-world experience and curated, personalised educational content.

## Conclusion

In this document, we have reviewed some of the use of digital slides for a range of learners. Digital slides represent a flexible, adaptable medium for histopathology education which can be tailored to support the needs of multiple synchronous or asynchronous learners, and can connect trainers and learners across geographical and temporal boundaries. Digital slides, supplemented with annotation software, yield a number of pedagogical advantages over glass slides, and provide a means for efficient integration of histopathology into undergraduate curricula, a support for postgraduate trainees, and a flexible approach to continuing professional education for senior clinicians. As digital slide technology becomes more prevalent in clinical practice, the role of the digital slide in postgraduate education will be augmented. Future educational tools and programmes incorporating virtual and augmented reality technology might build upon the innate versatility of the digital slide image.^4^
